# Molecular mechanisms of resistance and treatment efficacy of clofazimine and bedaquiline against *Mycobacterium tuberculosis*

**DOI:** 10.3389/fmed.2023.1304857

**Published:** 2024-01-10

**Authors:** Md Mahmudul Islam, Md Shah Alam, Zhiyong Liu, Mst Sumaia Khatun, Buhari Yusuf, H. M. Adnan Hameed, Xirong Tian, Chiranjibi Chhotaray, Rajesh Basnet, Haftay Abraha, Xiaofan Zhang, Shahzad Akbar Khan, Cuiting Fang, Chunyu Li, Sohel Hasan, Shouyong Tan, Nanshan Zhong, Jinxing Hu, Tianyu Zhang

**Affiliations:** ^1^State Key Laboratory of Respiratory Disease, Guangzhou Institutes of Biomedicine and Health, Chinese Academy of Sciences, Guangzhou, China; ^2^Guangdong-Hong Kong-Macao Joint Laboratory of Respiratory Infectious Diseases, Guangzhou Institutes of Biomedicine and Health, Chinese Academy of Sciences, Guangzhou, China; ^3^University of Chinese Academy of Sciences, Beijing, China; ^4^China-New Zealand Joint Laboratory on Biomedicine and Health, Guangzhou Institutes of Biomedicine and Health, Chinese Academy of Sciences, Guangzhou, China; ^5^Department of Microbiology, Shaheed Shamsuzzoha Institute of Biosciences, Affiliated with University of Rajshahi, Rajshahi, Bangladesh; ^6^Guangzhou Medical University, Guangzhou, China; ^7^Guangzhou National Laboratory, Guangzhou, China; ^8^Department of Medicine, Center for Emerging Pathogens, Rutgers-New Jersey Medical School, Newark, NJ, United States; ^9^Laboratory of Pathology, Department of Pathobiology, University of Poonch Rawalakot, Azad Kashmir, Pakistan; ^10^Department of Biochemistry and Molecular Biology, University of Rajshahi, Rajshahi, Bangladesh; ^11^State Key Laboratory of Respiratory Disease, Guangzhou Chest Hospital, Guangzhou, China; ^12^State Key Laboratory of Respiratory Disease, National Clinical Research Center for Respiratory Disease, The National Center for Respiratory Medicine, The First Affiliated Hospital of Guangzhou Medical University, Guangzhou, China

**Keywords:** cross-resistance, drug resistance, mechanism of action, molecular diagnostic, regimens, treatment

## Abstract

Clofazimine (CFZ) and bedaquiline (BDQ) are currently used for the treatment of multidrug-resistant (MDR) *Mycobacterium tuberculosis* (*Mtb*) strains. In recent years, adding CFZ and BDQ to tuberculosis (TB) drug regimens against MDR *Mtb* strains has significantly improved treatment results, but these improvements are threatened by the emergence of MDR and extensively drug-resistant (XDR) *Mtb* strains. Recently, CFZ and BDQ have attracted much attention for their strong clinical efficacy, although very little is known about the mechanisms of action, drug susceptibility test (DST), resistance mechanisms, cross-resistance, and pharmacokinetics of these two drugs. In this current review, we provide recent updates on the mechanisms of action, DST, associated mutations with individual resistance and cross-resistance, clinical efficacy, and pharmacokinetics of CFZ and BDQ against *Mtb* strains. Presently, known mechanisms of resistance for CFZ and/or BDQ include mutations within the *Rv0678*, *pepQ*, *Rv1979c*, and *atpE* genes. The cross-resistance between CFZ and BDQ may reduce available MDR-/XDR-TB treatment options. The use of CFZ and BDQ for treatment in the setting of limited DST could allow further spread of drug resistance. The DST and resistance knowledge are urgently needed where CFZ and BDQ resistance do emerge. Therefore, an in-depth understanding of clinical efficacy, DST, cross-resistance, and pharmacokinetics for CFZ and BDQ against *Mtb* can provide new ideas for improving treatment outcomes, reducing mortality, preventing drug resistance, and TB transmission. Along with this, it will also help to develop rapid molecular diagnostic tools as well as novel therapeutic drugs for TB.

## 1 Introduction

Tuberculosis (TB) is one of the most frequent chronic infectious diseases that poses an important threat to public health and a big global social problem (1) with 1.6 million deaths owing to TB in 2021 (2). One of the key reasons for deaths due to TB is drug resistance. The COVID-19 pandemic is still having a damaging affect for both TB diagnosis and treatment cases. Of note, the success rate of cure is very high for drug-sensitive patient using the two most-effective drugs isoniazid (INH) and rifampicin (RIF) compared to drug-resistant (DR) patient. The rapid increase of multidrug-resistant TB (MDR-TB, defined as combined resistance to RIF and INH) is a major challenge for treating and controlling of TB transmission worldwide. According to latest TB reports, close to half a million (450000) people developed MDR/ RIF-resistant TB (RR-TB) of which 3.6% among new cases and 18% among previously treated (2). Notwithstanding, pre-extensively drug-resistant TB (pre-XDR-TB) and XDR-TB raise more concerns for treating and preventing TB (1). The new definitions of pre-XDR-TB [defined as TB caused by *Mtb* strains that fulfill the definition of MDR/ RR-TB and are additional resistant to any fluoroquinolone (FQ) drug] and XDR-TB [defined as TB caused by *Mtb* strains that fulfill the definition of MDR/RR-TB and are additional resistant to any FQ drug plus at least one more drug of Group A, bedaquiline (BDQ) or linezolid (LZD)] have been introduced into TB program by the World Health Organization (WHO) in October 2021 and applicable from January 2021 (3, 4). The overall treatment success rates of MDR/RR-TB and XDR-TB were only 60 and 39%, respectively (1, 5). The treatment regimens for DR-TB, particularly for MDR-TB and XDR-TB, are longer, more expensive, more toxic, and less efficacious compare to drug-susceptible TB (DS-TB). However, the treatment of MDR-TB and XDR-TB has often failed owing to drug resistance and a lack of effective anti-TB drugs. Therefore, there is an urgent demand to develop novel anti-TB drugs with more effective, less expensive, and a short treatment cycle for the treatment of DR-TB.

Clofazimine (CFZ), a riminophenazine antimicrobial agent, is primarily used for the treatment of *Mycobacterium leprosy* infections. This drug is also recommended by the World Health Organization (WHO) as a key drug in both shorter and longer DR-TB regimens (6, 7). However, CFZ has good activity against DR *Mtb* strains for both *in vitro* and *in vivo* studies (8). The mechanism of action and resistance to CFZ are not fully understood (7). Mutation in *Rv0678* gene, encoding the MmpR5 repressor protein, is associated with resistance to CFZ in *Mtb* strains. In addition, mutations in the *Rv2535c* (*pepQ*) and *Rv1979c* genes have also been found in CFZ-resistant mutants (9).

Bedaquiline (BDQ), a diarylquinoline, was approved in 2012 by the Food and Drug Administration for the treatment of MDR-TB (10) and it inhibits the adenosine triphosphate (ATP) synthase encoded by *atpE* gene of *Mtb*. Cohort studies reported that success rates of MDR-TB treatment with BDQ-containing regimens was 70–80% (11–13). Similar results have been found for XDR-TB, where treatment results without BDQ are even worse (13, 14). The use of BDQ for the treatment of MDR-and XDR-TB reduces the mortality rate (15, 16). Therefore, the rapid appearance of BDQ resistance soon after introduction is alarming. Recently, BDQ resistance appeared in *Mtb* in both *in vitro* and clinical studies (17–19). Currently known resistance mechanisms to BDQ are associated with mutations within the *atpE*, *Rv0678*, and *pepQ* genes (20, 21). Significantly, mutations in the *atpE* which is the main pathway for BDQ cause higher-level of BDQ resistance and these mutations do not appear to impart cross-resistance to CFZ.

Cross-resistance is appeared between BDQ and CFZ due to confer mutations in the *Rv0678, Rv1979c*, and *pepQ* genes (22). The mutations in *Rv0678* gene have been reported in both MDR-and drug-sensitive *Mtb* strains from TB patients without a history of treatment with CFZ or BDQ (17, 23). Interestingly, CFZ-resistant spontaneous mutants harbored *Rv0678* mutations that confer cross-resistance to BDQ, indicating clinical use of CFZ could lead to BDQ resistance even without BDQ use (18). Unfortunately, there are limited clinical data, particularly in TB-epidemic regions, on the association for cross-resistance between genotypic and phenotypic BDQ and CFZ resistance in *Mtb* clinical isolates. The development of reliable drug susceptibility testing (DST) and genetic-based resistance screening for CFZ and BDQ are urgently needed. Therefore, this review is focused on providing recent updates on the mechanism of action, associated mutations with individual resistance and cross-resistance, DST, clinical uses, and pharmacokinetics to CFZ and BDQ against *Mtb* strains.

## 2 Clofazimine

CFZ, a riminophenazine antibiotic, was initially discovered in Dublin in the 1950s’ and used in the treatment of leprosy caused by *M. leprae* (24, 25). CFZ possesses both anti-inflammatory, pro-oxidative, and anti-mycobacterial properties (26, 27). Recently, this drug has gained attention once more as a substitute for treating *Mtb* infections (24, 28). Notably, CFZ has demonstrated potent activity against *Mtb*, including MDR-TB strains in both studies *in vitro* and in animal (8, 29–31). Additionally, the adverse effects of CFZ include cutaneous, gastrointestinal, and skin side effects, as well as a relatively low plasma drug concentration and a lengthy half-life (32). The demand of the CFZ-containing regimens for the treatment of MDR-TB increased after a study conducted in Bangladesh (33). Their observational study assessed the efficiency of standardized regimens with second-line drugs for untreated MDR-TB strains. The introduction of CFZ increased the success rate in MDR-TB treatment cohorts in clinical trials (8, 28, 34). Therefore, understanding the initial level of resistance is necessary to formulate a suitable strategy for the potential widespread use of CFZ against MDR-TB strains in developing nations.

## 3 Mechanism of action of CFZ

**Figure 1:**
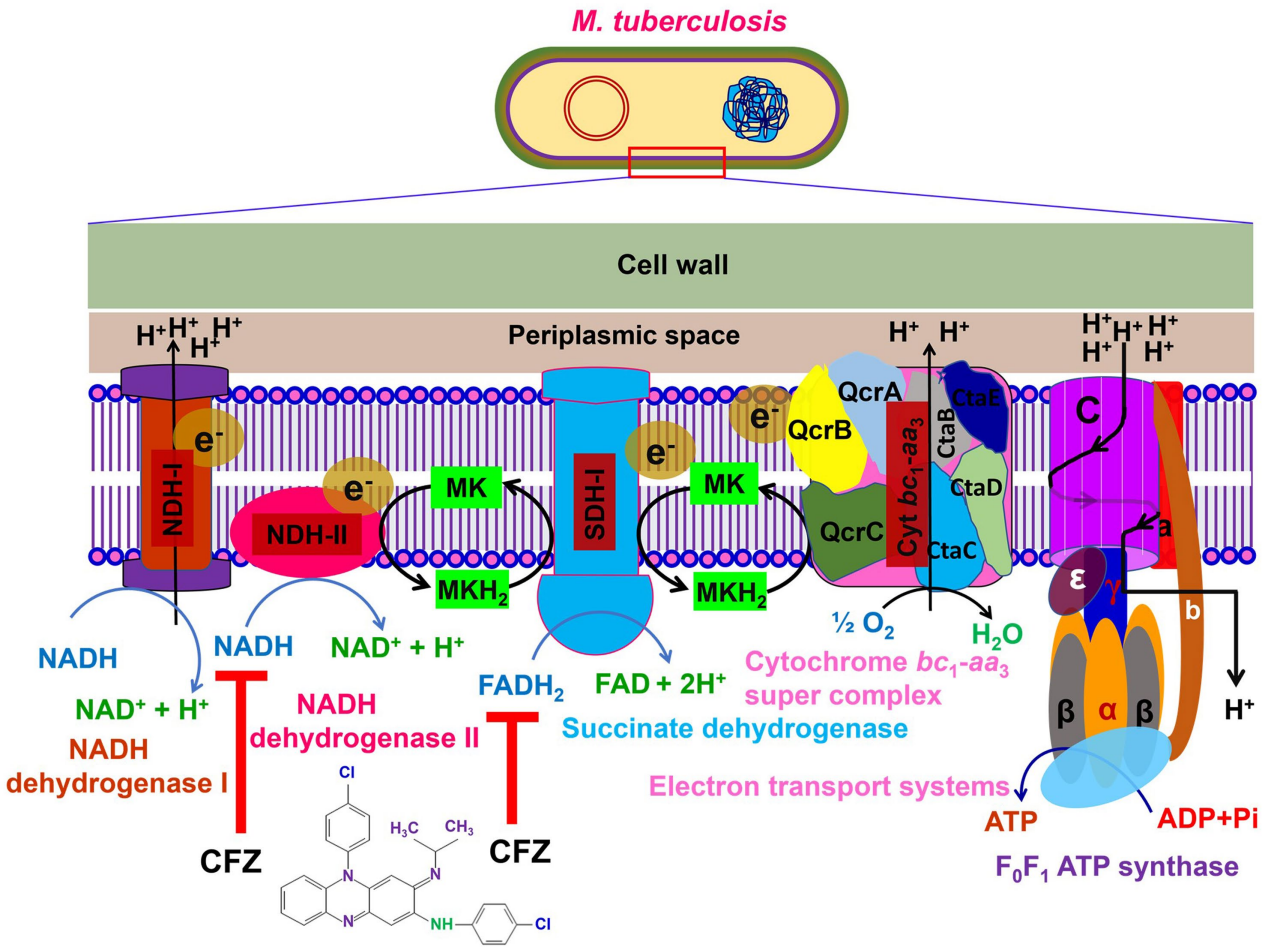
The mechanism of action of CFZ to the bacterial membrane. The electron transport chain (ETC) is a series of complexes for electron transfer from electron donors to electron acceptors across the bacterial membrane and shuttles electron from NADH and FADH_2_ to molecular O_2_. The protons are pumped out via electron movement from NADH and FADH_2_ then moves to ATP synthase for ATP production. So, CFZ accepts electron from the NADH dehydrogenase-II and thereby reduction flow of electrons through the mycobacterial ETC and reducing the synthesis of ATP production.

Currently, the mechanism of action of CFZ is still not well understood (7). It is reported that CFZ targets several sites in tubercle bacilli (35). However, it has been asserted that the cellular membrane of this antibiotic appears to be its primary site of action. The putative targets for the mechanism of action of CFZ include the mycobacterial respiratory chain and ion transporters (36, 37). In *Mtb*, CFZ seems to function like a prodrug that is reduced by NADH dehydrogenase (NDH-II) leading to reoxidation by oxygen (O_2_) to produce reactive oxygen species (ROS). Menaquinone (MK-4), a key component in the electron transfer chain (ETC) enzyme NDH-II of mycobacteria (36, 38), competes with CFZ for NDH-II reduction (Figure 1). Significantly, CFZ acts as a synthetic electron acceptor and thereby reduces flow of electrons through the mycobacterial ETC (7) and ultimately affects the generation of ATP. According to another theory, CFZ interacts with bacterial membrane phospholipids to produce antimicrobial toxic lysophospholipids. Then, lysophospholipids prevent potassium (K^+^) absorption and ultimately decreasing or inhibiting ATP production and thereby cause significant membrane instability (24). Other proposed that CFZ binds to the bacterial DNA for blocking the function of DNA and thereby inhibits bacterial proliferation (39).

Recently, CFZ has garnered attention in order to its ability anti-inflammatory properties in both infectious diseases and immune diseases including Crohn disease, discoid lupus erythematosus, ulcerative colitis, chronic lymphocytic leukemia, etc. (26). Notably, the pandemic status of the coronavirus disease of 2019 (COVID-19) was announced in March 2020. Since then, a number of investigations have been carried out to determine the best treatment care for this unique infection. CFZ was among the drugs that demonstrated promising outcomes against COVID-19 (40–42). Because it prevents T-lymphocyte activation and proliferation, CFZ has anti-inflammatory activity. A number of mechanisms have been hypothesized, including the direct inhibition of T-cell Kv 1.3 potassium channels, and indirect action by encouraging the release of E-series prostaglandins and ROS from nearby monocytes and neutrophils. In conclusion, CFZ appears to have a variety of pathways for fighting bacteria, may be with varied significance placed on various processes depending on the physiological conditions (24).

## 4 Drug susceptibility test of CFZ

The Clinical & Laboratory Standards Institute (Wayne, PA, United States) and United States Food and Drug Administration (Silver Spring, MD, United States) have not established a breakpoint for CFZ susceptibility testing, despite the fact that the WHO has based its estimate of the critical concentration (CC) in mycobacteria growth indicator tube (MGIT) at 1 mg/L on tiny research and unpublished data (32, 43–45). Previous research has shown that CFZ has *Mtb* minimal inhibitory concentrations (MICs), including MDR-TB strains, with typical ranges between 0.125 and 2.0 mg/L. (32, 46, 47) According to several studies, CFZ breakpoints were ranges between 0.25 and 1 mg/L. (44, 45, 48–50) The CCs or cutoff of CFZ for DST are summarized in Table 1. In a different Chinese investigation, 90 XDR-TB strains were used to assess the MIC using the microplate alamar blue assay (MABA) method against several drugs includes CFZ, and BDQ (48). They found the breakpoints MIC of CFZ for drug susceptibility were defined as 1.0 μg/mL. Finally, it can be concluded that the supporting laboratory and clinical data for CFZ resistance are required regarding the DST and MICs. On the other hand, the range of BDQ susceptibility is 0.12 to 2.0 μg/mL in different methods (51–56).

**Table 1:** The CC or cutoff of CFZ and BDQ for defined in the evaluated articles.

DST method	CC for CFZ (mg/L)	References	CC for BDQ (mg/L)	References
(Bactec) MGIT960	1.0	(32, 44, 45)	0.8 1.0 2.0	(21, 45, 51)
Resazurin microtiter assay (REMA)	1.0	(44)	0.125 0.25	(52, 53)
Agar proportion method (APM on 7H10 or 7H11)	1.0	(44)	0.12 0.25	(18, 54)
Broth Microdilution (BMD)	0.25 0.5	(49) (8)	0.25	(8, 49)
Microplate alamar blue assay (MABA)	1.0	(48, 50)	0.12 0.25	(48, 50, 55, 56)

CC, Critical concentration; CFZ, Clofazimine; BDQ, Bedaquiline; DST, drug susceptibility testing.

## 5 Clinical treatment efficacy of CFZ

Several new and repurposed oral anti-TB drugs include BDQ, pretomanid (PMD), delamanid (DLM), CFZ, LZD, and carbapenems (CPM), appear to be safe and effective enough to treat in a majority of MDR-and XDR-TB patients. CFZ has been used for leprosy treatment since 1962. The use of CFZ has been interested to treat MDR-TB owing to increasing emergence of MDR-*Mtb* strains (57, 58). CFZ-containing regimens have shown improved results in the treatment of RR-TB and MDR-TB cases. Most of the CFZ-containing shorter treatment regimen (STR) studies conducted in different countries, i.e., Bangladesh, Cameroon, Niger, Guinea, Africa, Vietnam, Burundi, China, and Uzbekistan etc. have demonstrated great therapeutic success (the total of cure and treatment completed) rates between 66.3 and 92.9% for RR-/MDR-TB patients (Table 2) (33, 34, 59–71).

**Table 2:** Summary of observational research studies describing for RR-/MDR-TB patients treated with CFZ-containing individual or standardized shorter treatment regimen.

Characteristics							Treatment outcomes							
Author (Year), References	Country	Study Population (Age of Patients)	Type of study	Treatment regimen used in combination with CFZ	Type of regimen	TD^m^	Favorable outcomes			Unfavorable outcomes				
							Cure	Completed	Total	Failure	Death	Default	Relapse	LTFU
Van Deun et al. (33)	Bangladesh	427 patients with MDR-TB	Prospective cohort	4*(Km + Cfz + Gfx + E + Hh+ Z + Pto) for inten. and 5 (Gfx + E + Z + Cfz) for con.	STR	9–12	170/206 (82.5%)	11/206 (5.3%)	181/206 (87.9%)	1/206 (0.5%)	11/206 (5.3%)	12/206 (5.8%)	1/206 (0.5%)	-
Aung et al. (59)	Bangladesh	515 patients with MDR-TB	Prospective cohort	4*(Km + Cfz + Gfx-h + E+ Hh + Z + Pto) for inten. and 5(Gfx + E + Z + Cfz) for con.	STR	9–12	418/515 (81.2%)	17/515 (3.3%)	435/515 (84.5%)	7/515 (1.4%)	29/515 (5.6%)	40/515 (7.8%)	4/515 (0.8%)	-
Piubello et al. (60)	Niger	65 patients with MDR-TB	Prospective cohort	4^+^(Km + Cfz + Gfx-h + E+ Hhm + Z + Pto) for inten. and 8(Gfx + E + Z + Cfz) for con.	STR	12–14	58/65 (89.2%)	0	58/65 (89.2%)	-	6/65 (9.2%)	1/65 (1.6%)	-	-
Kuaban et al. (61)	Cameroon	236 patients with MDR-TB	Prospective cohort	4^+^(Km + Cfz + Gfx + E + H+ Z + Pto) for inten. and 8(Gfx + E + Z + Cfz + Pto) for con.	STR	12–14	132/150 (88.0%)	2/150 (1.3%)	134/150 (89.3%)	1/150 (0.7%)	10/150 (6.7%)	5/150 (3.3%)	-	-
Tang et al. (62)	China	105 patients with MDR-TB	Prospective	NA	Individual	21	27/53 (50.9%)	12/53 (22.6%)	39/53 (73.6%)	6/53 (11.3%)	4/53 (7.5%)	4/53 (7.5%)	-	-
Trébucq et al. (63)	9 African countries	1,006 patients with MDR-TB	Prospective cohort	4^+^(Km + Cfz + Mfx + E + H-h + Z + Pto) for inten. And 5 (Mfx + E + Z + Cfz) for con.	STR	9–12	728/1006 (72.4%)	93/1006 (9.2%)	821/1006 (81.6%)	59/1006 (5.9%)	78/1006 (7.8%)	-	-	48/1006 (4.8%)
Duan et al. (64)	China	156 patients with MDR-TB	Randomized trial	24(Cfz + Lfx + Z + E + Pas/Pto + Amx/Clv)	Individual	24	36/66 (54.5%)	7/66 (10.6%)	43/66 (65.1%)	9/66 (13.6%)	4/66 (6.1%)	10/66 (15.2%)	-	-
Du et al. (34)	China	135 patients with MDR-TB	Prospective cohort	6(Cm + Cfz + Cs + Lfx + Pto+ Z) for inten. and 6(Cfz+ Cs + Lfx + Pto + Z) for con.	STR	12	42/67 (62.7%)	4/67 (6.0%)	46/67 (68.7%)	7/67 (10.4%)	2/67 (3.0%)	12/67 (17.9%)	-	-
Anh et al. (65)	Vietnam	302 patients with RR/ MDR-TB	Cohort	4^+^(Lfx + Km + Cfz + Pto + E + Hh + Z) for inten. And 5(Lfx + Cfz + E + Z) for con.	STR	9–12	246/302 (81.5%)	13/302 (4.3%)	259/302 (85.8%)	16/302 (5.3%)	13/302 (4.3%)	-	-	14/302 (4.6%)
Hassane-Harouna et al. (66)	Guinea	271 patients with RR-TB	Retrospective cohort	4^+^(Km + Mfxh+ Pto + Hh+ Cfz + E + Z) for inten. and 5(Mfxh+Cfz + E + Z) for con.	STR	9–12	112/196 (57.1%)	33/196 (16.8)	145/196 (74%)	5/196 (2.6%)	30/196 (15.3%)	-	-	16/196 (8.2%)
Ciza et al. (67)	Burundi	225 patients with RR-TB	Retrospective cohort	4^+^(Km + Cfz + Mfx + E + H-h + Z + Pto) for inten. and 5 (Mfx + E + Z + Cfz) for con.	STR	9	185/225 (82.2%)	25/225 (10.7%)	209/225 (92.9%)	1/225 (0.4%)	11/225 (4.9%)	-	1/225 (0.4%)	3/225 (1.3%)
Souleymane et al. (68)	Niger	195 patients with RR-TB	Retrospective cohort	NA	STR	9–11	161/195 (82.6%)	0	161/195 (82.6%)	7/195 (3.6%)	24/195 (12.3%)	-	-	3/195 (1.5%)
Trubnikov et al. (69)	Uzbekistan	95 patients with RR/MDR-TB	Cohort	4^+^(Mfx + Cm + Cfz + Pto + Z + E + Hh) for inten. and 5(Mfx + Cfz + Pto + Z + E) for con.	STR	9–12	63/95 (66.3%)	0	63/95 (66.3%)	17/95 (17.9%)	7/95 (7.4%)	-	-	5/95 (5.3%)
du Cros et al. (70)	Uzbekistan	146 patients with MDR-TB	Prospective cohort	4^+^(Z + E + Hh + Mfx + Cm or Km + Pto + Cfz) for inten. and 5(Z + E + Mfx + Pto+ Cfz) for con.	STR	9–12	55/128 (43.0%)	37/128 (28.9%)	92/128 (71.9%)	22/128 (17.2%)	2/128 (1.5%)	-	-	16/128 (12.5%)
Yao et al. (71)	China	68 patients with MDR-TB	Prospective, randomized, controlled	6(Bdq + Lfx + Lzd + Cs + Cfz) for inten. and 12(Lfx + Lzd +Cs + Cfz) for con.	STR	18	28/34 (82%)	0	28/34 (82%)	5/34 (15%)	-	-	-	1/34 (3%)

TB, Tuberculosis; RR, Rifampicin-resistant; MDR, Multidrug-resistant; Km, Kanamycin; Cm, Capreomycin; Cfz, Clofazimine; Gfx, Gatifloxacin; Gfx-h, High-dose gatifloxacin; E, Ethambutol; H, Isoniazid; Hh, High-dose isoniazid; Hhm, Medium-high dose isoniazid; Z, Pyrazinamide; Pto, Prothionamide; Mfx, Moxifloxacin; Mfxh, High-dose moxifloxacin; Lfx, Levofloxacin; Cs, Cycloserine; Amx, Amoxicillin; Clv, Clavulanate; NA, Not available; LTFU: Lost to follow-up; TD^m^, Treatment duration (months). *The intensive phase was prolonged if the results of the sputum smear microscopy at month 4 were positive until the results were negative or the patient was deemed to have failed the bacteriological treatment. ^+^The intensive phase was prolonged up to 6 months in the case of delayed sputum smear conversion at 4 months.

In Bangladesh, Van Deun and his team searched a shorter and more efficacies treatment regimen for MDR-TB to increase treatment success rate and to minimize treatment failure, death and lost to follow-up (33). They found a standardized 9-month Bangladesh regimen after evaluating six combinations of drugs and duration of treatment. The 9-month Bangladesh regimen includes gatifloxacin (GFX) in combination with CFZ, ethambutol (EMB), and pyrazinamide (PZA) throughout, supplemented by kanamycin (KAN), prothionamide (PTO), and high-dose isoniazid (INHh) during an intensive phase of 4 months (4–6 GFX-CFZ-EMB-PZA-KAN-PTO-INHh−/5 GFX-CFZ-EMB-PZA) and this phase was increased up to maximum 6 months, keeping the continuation phase duration at 5 months. Significantly, in 206 patients those received this STR treatment, the relapse-free cure rate was 87.9% (95% confidence interval, 82.7–91.6). In 2014, almost similar outcomes for 515 patients were reported by the same research team from Bangladesh (59). These findings were further supported those reported from China (82%), Niger (82.6%), Vietnam (85.8%), Cameroon (89.3%), Burundi (93%) and these results are highly effective and well tolerated against MDR-TB but not previously exposed or resistance to second-line drugs (61, 65, 67, 68, 71).

A recent interesting prospective, randomized, multicenter study conducted in China was carried to compare between 12-month treatment CFZ-containing shorter regimen and 18-month without CFZ-containing regimen for MDR-TB patients. Significantly, 68.7% MDR-TB patients receiving CFZ-containing shorter regimen had sputum culture conversion in comparison with 55.9% of those receiving regimen without CFZ, reflecting an early culture conversion (*p* = 0.04). This finding indicates that CFZ-containing shorter regimen had a comparable successful result when compared to without CFZ-containing regimen. The patients assigned to the CFZ-containing shorter regimen showed more rapid culture conversion compared to without CFZ-containing regimen, indicating impressive antimicrobial activity against MDR-TB (34). Another prospective, randomized, multicenter, controlled, and open study conducted in same country (China) was done 21-month of individual-based treatment regimens to compare between CFZ-containing group and control (without CFZ) group against MDR-TB. The treatment successful outcome rate of this study in the CFZ group was 73.6% which is significantly higher than that in control (without CFZ) group (53.8%; *p* = 0.035). The adverse effect in skin only found in the CFZ-containing group (62). A very recent study in West and Central Africa stated CFZ-containing a nine-month short regimen for the treatment of RR-TB cases. The overall treatment success rate of this short regimen (4–6 KAN + CFZ + MFX + EMB + INHh+PZA + PTO/5 MFX + EMB + PZA + CFZ) was close to 80% relapse-free cure rate among 1,006 patients, which indicates the good outcome in low-and middle-income settings against RR-TB cases (72). Similarly, a recent meta-analysis showed greater favorable treatment outcomes after the use of CFZ-containing regimen compared with those receive no CFZ against MDR-TB cases (73). In 2018, another previously published meta-analysis reported that CFZ-containing regimens were associated with significantly improved treatment outcomes for MDR-TB (74).

For the CFZ-containing STR against RR-/MDR-TB cases, the fluoroquinolones (FQs) including GFX, moxifloxacin (MFX), and levofloxacin (LFX) are the core drugs. The activity of high-dose GFX-based regimens is higher than that of high-dose LFX-based or normal-dose MFX-based regimens (75, 76). The GFX-based STR was highly effective (approximately >84% treatment success rate) among MDR-TB cases in Bangladesh, Niger and Cameroon (77). Significantly, low-level drug resistance can be overcome when high-dose GFX is used (75, 76). GFX is not currently included in most STR setting programs to treat MDR-TB. These findings suggest reintroducing GFX into STR against RR-/MDR-TB treatment programs (75, 77).

It is very important to note that CFZ-containing STR is used second-line injectable drug (SLID), i.e., KAN or capreomycin (CM), to prevent acquired FQs resistance during the 4–6 months intensive phase. However, drug adverse event ototoxicity induced by the SLID used in the intensive phase is the primary concern ranging between 3.2 and 32.6% (67, 69, 78, 79). In a very recent meta-analysis by Wrohan et al. (80) reported that the incidence of hearing loss was 28.3% of MDR-TB patients receiving SLID after analysis of 64 studies from different 25 countries including 12,793 patients. The drug adverse ototoxicity due to receiving SLID can be permanent and may continue even after stopping these medications. The WHO is not recommending to enabling access any SLID to STR for the treatment of patients with RR-/MDR-TB due to severe side effects. Therefore, in 2020, the WHO is urging all countries the use of fully-oral treatment regimens for RR-/MDR-TB patients, either short or long (6). Although the efficacy of fully-oral treatment regimens are still not well understood. Importantly, a retrospective cohort study has shown that BDQ can effectively replace the SLID (81). Notwithstanding, many studies are still needed to assess the safety and efficacy of fully-oral treatment regimens for patients with RR-, MDR-/XDR-TB.

For XDR-TB, a multicenter, randomized, and prospective study investigated 36-months of individual-based treatment regimen to find out the efficacy and safety after treatment with CFZ, compared with XDR-TB patients those received no CFZ. They found CFZ-containing individual-based regimen did not increase the favorable treatment results or shorten the culture conversion time, when compared with no CFZ-containing regimen against XDR-TB patients (82). On the other hand, the treatment of XDR-TB showed that CFZ-containing regimens were more effective with cure rates 40% compared with receiving a non-CFZ regimen cure rate 28.6%. Adverse effects due to CFZ were infrequent and rarely serious enough to be life-threatening (83). We advise empirical inclusion of CFZ in XDR-TB therapy regimens because to the current low rates of culture conversion.

## 6 Resistance mechanisms of CFZ

CFZ has recently been demonstrated a good therapeutic effect for the treatment of MDR-TB and to shorten TB treatment. Despite the significance of CFZ in different regimens, programmatic implementation attempts have been hampered by the length and safety of these regimens, both of which are factors that encourage the emergence of resistance. Of note, less than 40% of CFZ-resistant strains had changes in genes known to induce CFZ resistance, which is in line with this study and suggests that additional research should be done to understand how CFZ works (8). The resistance to CFZ was detected in 8.3% (23/277) of the MDR-TB isolates. It is significant to note that the rate of acquired resistance to CFZ (6.3%, 12/189) was noticeably higher than the rate of primary resistance (12.5%, 11/88, *p* = 0.028). According to these findings, MDR-TB patients in China showed a significant prevalence of CFZ resistance. Another similar reported a prevalence of 7.4% (29/391) from patients of MDR-TB in South Africa (21). CFZ was not frequently utilized for MDR-TB therapy in the Chinese population due to an increased prevalence of skin discoloration. As a result, the high level of CFZ resistance raises additional worries about the potential of high mutation rate following exposure to the treatment. Notably, a current study by Shang et al. (50) reported 12/13 (92.31%) CFZ-resistant isolates were resistant to BDQ when a MIC of CFZ was ≥4 mg/L. One of the main risk factors for associated BDQ resistance is pre-XDR, together with exposure to CFZ. The percentage of DR-*Mtb* isolates having CFZ resistance with MIC >1 mg/L was 4.1% in a recent study (32). Of note, this study also reported that XDR isolates have a higher rate of CFZ resistance than MDR isolates.

**Figure 2:**
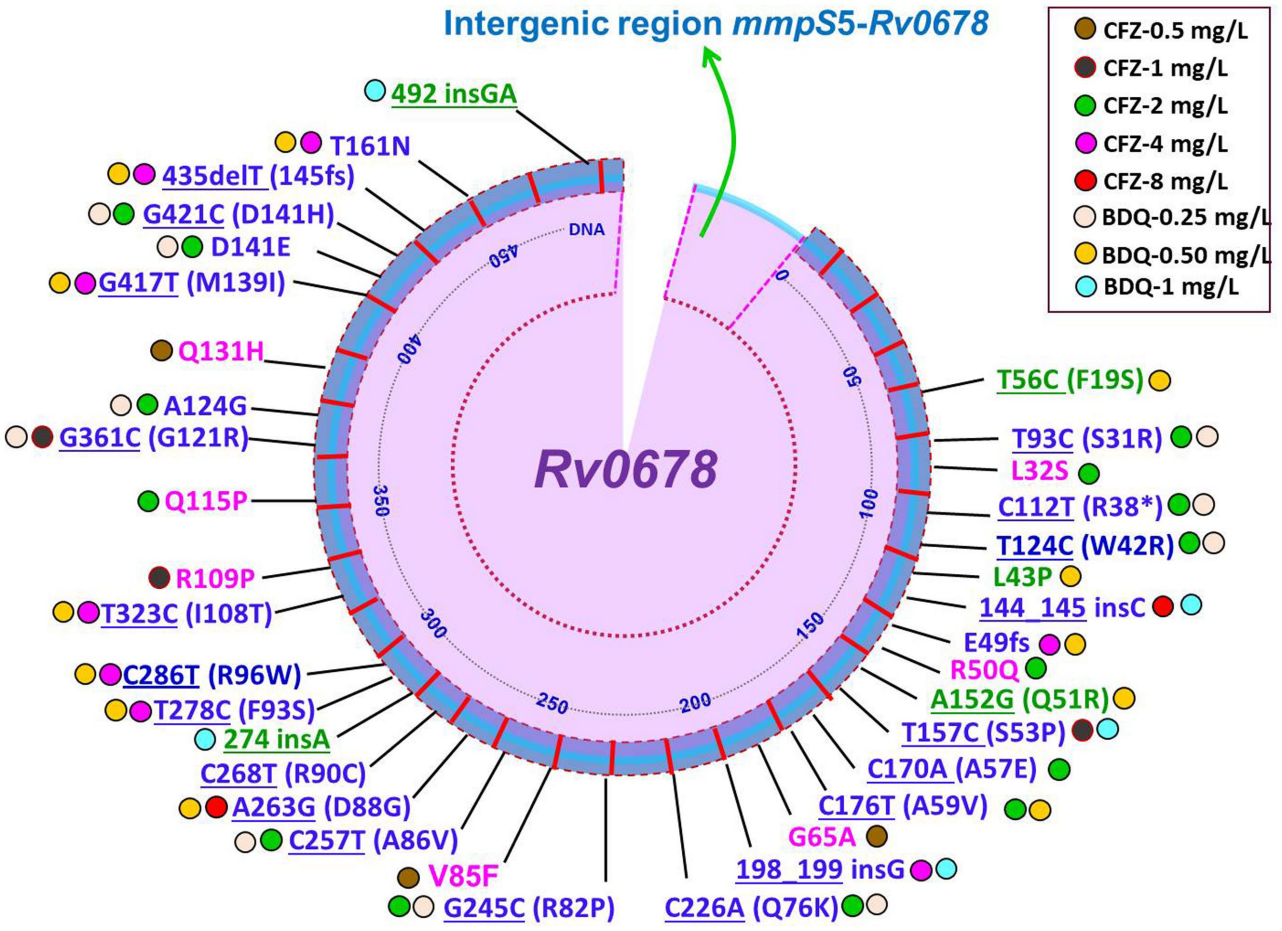
Schematic illustration of recently identified mutations in *Rv0678* associated with individual resistance or cross-resistance to BDQ and CFZ in *Mtb* clinical isolates. Blue color mutations indicated cross-resistance; Magenta color mutations indicated resistance only for CFZ; Green color mutations indicated resistance only for BDQ; Underline mutations indicated DNA and without underline indicated amino acids; *indicated stop codon (8, 22, 50, 55, 56, 87, 88, 90, 91).

**Figure 3:**
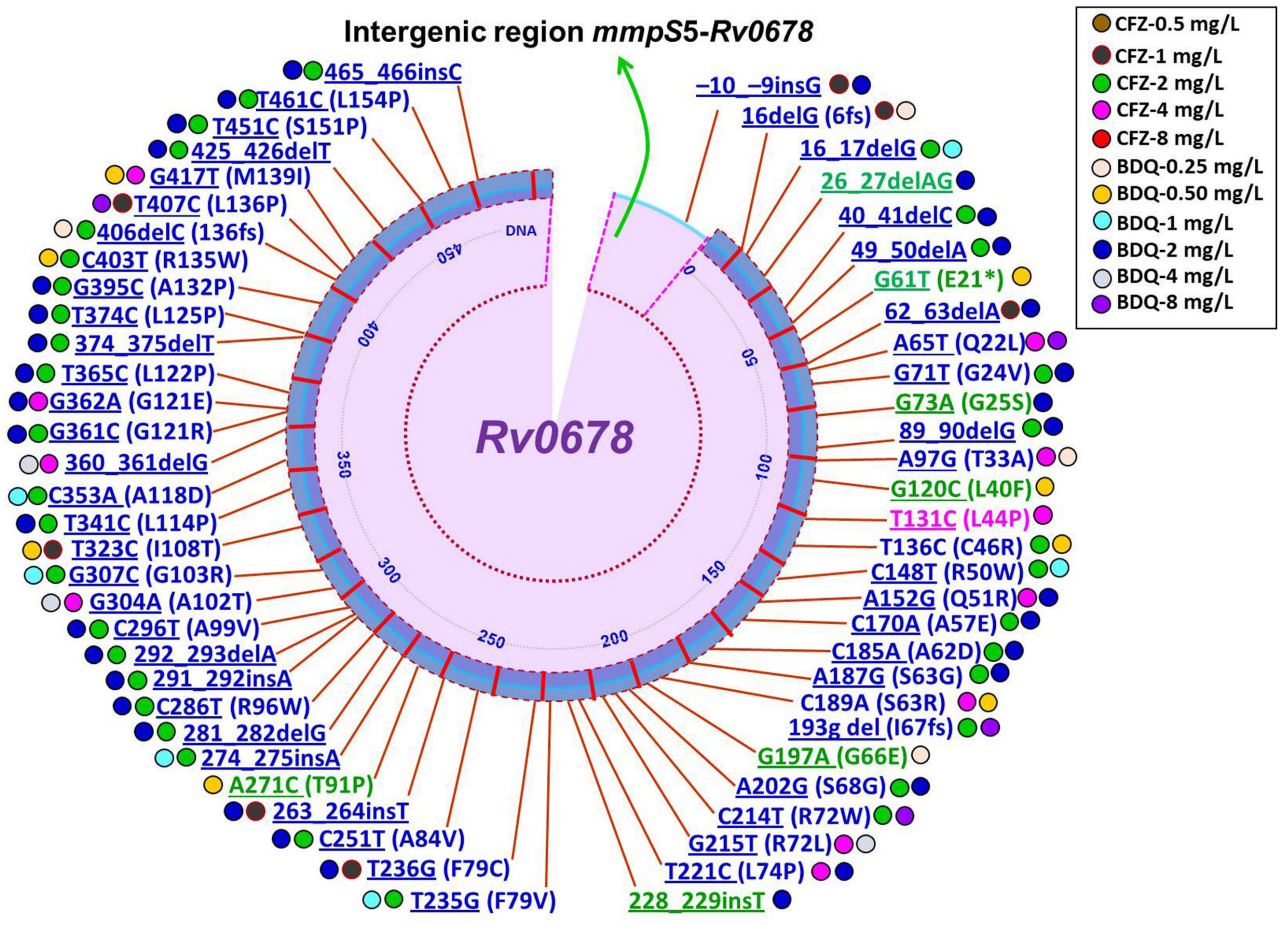
Schematic illustration of recently identified mutations in *Rv0678* associated with individual resistance or cross-resistance to BDQ and CFZ in *Mtb in vitro* and *in vivo* isolates. Blue color mutations indicated cross-resistance; Magenta color mutations indicated resistance only for CFZ; Green color mutations indicated resistance only for BDQ; Underline mutations indicated DNA and without underline indicated amino acids; * indicated stop codon (16, 18, 91–93).

It is still not completely understood how CFZ resistance develops. Mutations in *Rv0678, Rv1979c,* and *Rv1453* have been documented to be linked resistance to CFZ (9, 18, 32, 84, 85). A recently report demonstrated mutations in *Rv1979c*, *Rv1453* and/or *Rv0678* were present in only 40.2% (29/72) CFZ-resistant *Mtb* isolates (50). Interestingly, four of the seven CFZ-resistant *Mtb* isolates carried only the *Rv1979c* mutation, while the other three isolates carried *Rv0678* or *Rv1453* mutations. The four CFZ-resistant isolates, however, only had a mutation in *Rv1979c*, which had a comparatively low-level MIC to CFZ. Another similar study reported that mutations were harbored in *Rv1979c* and *Rv0678* in CFZ-resistant *Mtb* isolates accounted for 15.4% (86). One the other hand, several studies have revealed no mutation in *Rv1979c* was found among the CFZ-resistant strains (32). Current and several prior findings indicate that while the mutation in *Rv0678* did not the primary cause CFZ resistance in *Mtb* isolates, it did play a major role in generating the cross-resistance between BDQ and CFZ (8, 50, 87). The huge number of variant types as well as scattered, and lack of a clear hotspot characterization of *Rv0678* mutations were shown in a recently published review and research on CFZ resistance (32, 88, 89). Conversely, Zhang et al. (9) depicted at positions 193 and 466 of nucleotides in *Rv0678*, two mutational hot sites accounted for 42/96 (43.8%) and 11/96 (11.5%) of the mutations. Of note, different types of mutations in the *Rv0678* gene conferring resistance to CFZ derived clinical and *in vitro* isolates of *Mtb* are summarized in Figures 2, 3 (8, 18, 22, 50, 87, 88, 90–93). Finally, it can be concluded that phenotype and genotype tests are needed of CFZ in RR-/MDR-TB patients before considering as CFZ-containing regimen due to increasing resistance for inappropriate use.

## 7 Pharmacokinetics of CFZ

There has not been enough research done on the pharmacokinetics of CFZ, and data from TB patients are particularly rare. CFZ is incredibly lipophilic and abundantly distributes throughout fatty tissues. Because of its high lipophilicity, CFZ accumulates heavily in fat tissues while having relatively low blood concentrations (0.7–1 mg/L) (24, 94). The macrophage-rich organs of the lungs, liver, brain, spleen, and bone marrow are included in the fat tissues (95, 96). Whether a drug is administered along with or without food can have a significant impact on how well it is absorbed in human. CFZ is varied in its absorption after oral administration, ranging from 45 to 62% based on whether or not the CFZ is taken with food (24). Ingestion of food simultaneously increases the rate of CFZ absorption. The unaltered plasma peak is attained 6 to 12 h after a single oral dose of CFZ in the form of a capsule. The peak plasma concentration was 0.41 mg/L and the time to C_max_ was 8 h when a 200 mg CFZ tablet was ingested with food, on the other hand the peak plasma concentration was 30% lower and the time to C_max_ was 12 h when a 200 mg CFZ tablet was administered without food (24). Swanson and colleagues (96) reported that no variations in bacterial killing were seen between any of the CFZ doses given to *Mtb*-infected mice and the lowest dose was equally effective as the maximum dose. They suggested that it is possible to treat TB with considerably lower doses because the anti-TB activity of CFZ was not affected by either the dose given or the drug concentrations in the tissues. On the other hand, recently a study from South Africa depicted the body fat percentage had a significant impact on CFZ disposition, which led to reduced plasma exposure in women (97). They reported CFZ may need dose individualization at variations of body composition to maximize utilization, although the therapeutic effects are unknown (97, 98). Of note, it is stated that CFZ is more effectively absorbed when consumed with a high-protein and fat diet. On the other hand, CFZ will decrease its bioavailability when consumed with orange juice and an antacid (99).

The body retains CFZ for a long time. Therefore, the toxicities such as skin discoloration, QT prolongation and elevated liver enzymes are associated with CFZ, while it is not clear how these side effects relate to dose or plasma concentrations of the drug (100). The fact that CFZ causes the QT prolongation raises concerns because numerous other drugs, including BDQ, FQs, and DLM, that are approved by the WHO for treating DR-TB, also cause the QT interval to lengthen (101). The QT prolongation of CFZ is associated with cardiac arrhythmias. Therefore, the combination of three drugs includes MXF, BDQ, and CFZ that significantly lengthen the QT interval should not be used together in a regimen for TB treatment.

## 8 Bedaquiline

BDQ, a novel diarylquinolone drug, demonstrated outstanding efficacy against both DR- and DS-TB (55, 90). BDQ is now an essential component of the shorter oral regimen for MDR-TB treatment (102, 103). Significantly, the United States FDA granted rapid approval the combined use of BDQ and DLM for the treatment of MDR-/XDR-TB (37). The WHO in 2018 has recommended for using BDQ as an important antibiotic to be used along with LZD and FQs to treat MDR-TB (43). Surprisingly, a three-drug regimen consisting of LZD, BDQ, and PMD was evaluated with MDR-/XDR-TB patients in a recent study called the NIX-TB trial and the treatment was effective for 90% of TB patients (15). The use of BDQ is now spreading quickly and more than 90 countries having started using BDQ. However, inadequate knowledge of resistance mechanisms is impeding quick molecular diagnosis.

## 9 Mechanism of action of BDQ

**Figure 4:**
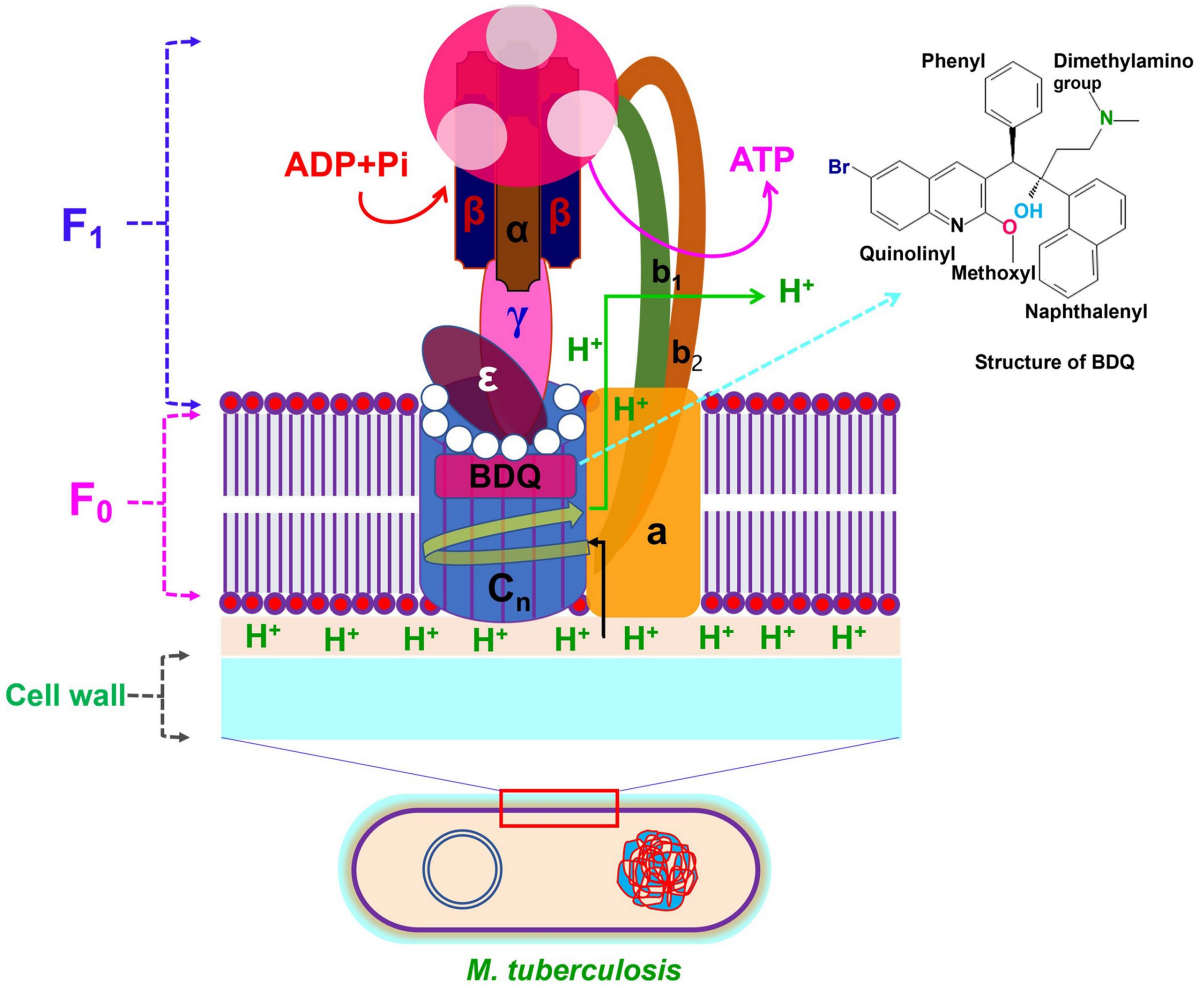
Structure of F_0_F_1_-ATP synthase with BDQ binding site. The F_0_ domain of ATP synthase is made of a, b_2_ and c*_n_* (*n* = 10–14) subunits. The F_1_ domain of ATP synthase is consist of 8 subunits including 3α, 3β, γ, δ and ε. BDQ binds to the subunit of c in the F_0_ rotor ring of the ATP synthase and inhibit the synthesis of ATP production.

BDQ is closely linked to FQs and chloroquine, albeit it has a distinct side-chain moiety. The mechanism of action of BDQ is different from FQs, and it has no inhibitory effects on DNA gyrase. Energy metabolism enzymes in *Mtb* for the development of drugs/compounds, such as F_0_F_1_ (F_0_, a rotor and F_1_, a stator) and the respiratory chain complexes, have been proved as new promising targets. Among the class of bioenergetics inhibitors, BDQ was the first drug approved by the United States FDA and the European Medicines Agency (103, 104). According to reports, BDQ interacts with the F_0_ domain to specifically target F_0_F_1_ of *Mtb* (105). As a result, ATP generation is inhibited, and ATP levels drop significantly. The ATP synthase enzyme consists of two sectors: the membrane sector F_0_, which contains three subunits (a, b_2_ and c*_n_*), and the cytoplasmic sector F_1_, which has five subunits (3α, 3β, γ, δ, and ε). The subunits c of F_0_ are organized in the shape of disks and serve as an ion-conducting route, while ADP and phosphate (Pi) are combined at three catalytic sites in F_1_ to generate ATP (104). It is widely known that BDQ has the ability to attach the subunit c in the F_0_ rotor ring of the ATP synthase and inhibit its function (Figure 4). In addition, BDQ has also been showed to prevent ATP synthesis via a second targeting binding site in the ε subunit on the mycobacterial F_0_F_1_-ATP synthase and prevent to generate ATP (106).

## 10 Drug susceptibility test of BDQ

BDQ has been given accelerated/conditional authorization for usage based on phase-II trials conducted in the European Union (2014), the United States (2012), and seven other nations with prevalence of MDR-TB incidence. Nowadays, BDQ is an important drug for the treatment of MDR-TB because it is included in the revised definitive WHO criteria for XDR-TB in 2021 (107). Two phase-II clinical investigations for BDQ used the preliminary DST approach that had previously been evaluated. The CC/cutoff values reported for BDQ susceptibility are summarized in Table 1 (8, 18, 21, 45, 48–56). Quality control (QC) parameters have been established in a multilaboratory, multicountry study for the BDQ phenotype DST utilizing 7H10 and 7H11 agar proportion method (APM) and 7H9 broth microdilution (BMD) MIC techniques (108). BDQ DST techniques and QC of MIC ranges against the *Mtb* H37Rv reference strain have been developed 0.015 to 0.06 μg/mL for the 7H9 BMD and 0.015 to 0.12 μg/mL for the 7H11 and 7H10 APM. Importantly, Keller et al. (109) reported BDQ DST using the MGIT 960 method and epidemiological cutoff value of 1.6 μg/mL, on the other hand, another study by Torrea et al. (110) proposed 1.0 μg/mL. Of note, a very recent systemic study by Nieto Ramirez et al. (111) reported that the most popular DST technique for BDQ was MGIT960. The CC or cutoff for BDQ susceptibility confirmed by several studies is 0.25 μg/mL for the 7H11 and 7H10 APM, 0.25 μg/mL for the BMD and 1.0 μg/mL for the MGIT 960 (43, 111, 112). Two studies for the resazurin microtiter assay (REMA) technique indicated a CC for BDQ of 0.25 mg/L, which was similarly obtained when utilizing BMD and MABA methods (53). Notably, the MIC susceptible breakpoint concentration was defined as ≤0.12 μg/mL by another study for BDQ when using MABA method (56), while another very recent study from China reported the breakpoint concentrations of BDQ susceptibility were defined as 0.25 μg/mL (55). Finally, it can be said that it is crucial to assess the MIC of BDQ even in patients those have no prior exposure to the drug or CFZ exposure when selecting an effective therapeutic regimen.

## 11 Clinical treatment efficacy of BDQ

Novel therapeutic anti-TB drugs like BDQ, DLM, LZD, and PMD are promising for the treatment of MDR, pre- and XDR-TB patients. The BDQ, an anti-TB drug, has been approved for the treatment of MDR-TB, and recommended for 24 weeks duration (11). Most of the BDQ-containing treatment studies conducted in different countries, i.e., France, Armenia, Georgia, Congo, South Africa, China, Nigeria, India etc., have demonstrated great therapeutic success (the total of cure and treatment completed) rates between 58.5 and 93.5% (Table 3) (11, 12, 14, 15, 27, 113–122).

**Table 3:** Summary of observational research studies describing for RR-/MDR-TB patients treated with BDQ-containing individual or standardized shorter/longer treatment regimen with/without CFZ drug.

Characteristics								Treatment outcomes						
Author (Year), References	Country	Study design	Study period	Study population (Age of patients)	HIV-positive	Treatment regimen used in combination with BDQ	TDT	Favorable outcomes (%)			Unfavorable outcomes (%)			
								Cure	Treatment completed	Total	Failure	Death	LFT	Default
Borisov et al. (12)	15 countries	Retrospective cohort	2008–2016	428 MDR and XDR	94/425 (22.1%)	Bdq, Lzd, Mfx, Cfz, Cpm	18 months (10–22 months)	154/247 (62.4%)	22/247 (8.9%)	176/247 (71.3%)	19/247 (7.7%)	33/247 (13.4%)	1/247 (0.4%)	18/247 (7.3%,)
Guglielmetti et al. (11)	France	Retrospective cohort	2011–2013	45 patients with MDR	2/45 (4.4%)	Bdq, E, Z, Cfz, Am, Cap, Eto, Mfx, Lfx, Cs, Ipm, Mpm, etc.	361 days	34/45 (75.6%)	2/45 (4.4%)	36/45 (80.0%)	1/45 (2.2%)	3/45 (6.7%)	5/45 (11.1%)	–
Hewison et al. (113)	Armenia and Georgia	Retrospective cohort	2013–2015	82 patients with MDR-TB/pre-XDRTB/XDR-TB (31–51 years)	4/82 (5.0%)	Bdq, Cfz, Lzd, Ipm	20–24 months	36/82 (43.9%)	12/82 (14.6%)	48/82 (58.5%)	6/82 (7.3%)	10/82 (12.2%)	18/82 (21.9%)	–
Ndjeka et al. (114)	South Africa	Retrospective cohort	2013–2015	200 patients with MDR and XDR	134/200 (67.0%)	Bdq, Cfz, Km, Lfx, Lzd	24 weeks	139/200 (69.5%)	7/200 (3.5%)	146/200 (73.0%)	9/200 (4.5%)	25/200 (12.5%)	20/200 (10.0%)	-
Olayanju et al. (14)	South Africa	Prospective cohort	2008–2017	272 patients with XDR-TB (18–73 years)	134/272 (49.3%)	Bdq, Cm, Km, Pas, Z, Trd, Mxf, Lfx, Lzd, E, Eto, Hh, Cfz, Amx-Clv, Mpm	24 months	45/68 (66.2%)	–	45/68 (66.2%)	4/68 (5.9%)	10/68 (14.7%)	8/68 (11.8%)	1/68 (1.5%)
Conradie et al. (15)	South Africa	Nix-TB	2015–2017	109 patients with MDR-TB/XDR-TB (17–60 years)	56/109 (51.4%)	Bdq, Pmd, Lzd	26–39 weeks*^a^*	98/109 (90%)	–	98/109 (90%)	–	7/109 (6.4%)	1/109 (0.9%)	0/109 (0.0%)
Padayatchi et al. (115)	South Africa	Retrospective cohort	2014–2015	194 patients with DR-TB/MDR-TB (27–41 years)	116/151 (76.8%)	Bdq, Lzd, Mxf/Lfx, Pas, Z, Cfz, Trd/Cs	24 months	95/151 (62.9%)	1/151 (0.7%)	96/151 (63.6%)	7/151 (4.6%)	26/151 (17.2%)	–	–
Kashongwe et al. (116)	Congo	Retrospective	2018	236 patients with MDR-TB/pre-XDR-TB	1/12 (8.3%)	Bdq, Km, Pas, Lzd, H, Cfz, Z, Dlm, Lfx, Mpm-Clv, Am, Ipm-clv, Cs	20 months	8/12 (66.6%)	2/12 (16.7%)	10/12 (83.3%)	–	1/12 (8.3%)	1/12 (8.3%)	–
Shi et al. (117)	China	Retrospective cohort	2018–2019	640 patients with MDR-TB/Pre XDR-TB/XDR-TB with or without DM	NS	Bdq, Mfx, Lfx, Lzd, Cfz, Cs, E, Z, Pto, Am, Cm, PAS, PH, Hh, Amx/Clv, Str, Clr	24 weeks	*66/107 (61.7%)	31/107 (29.0%)	97/107 (90.7%)	–	0/107 (0.0%)	–	3/107 (2.8%)
								^+^54/107 (50.5%)	46/107 (43.0%)	100/107 (93.5%)	–	0/107 (0.0%)	–	2/107 (1.9%)
Li et al. (118)	China	NA	2018–2020	35 patients with MDR-TB/pre-XDRTB/XDR-TB (19–73 years)	0/35 (0.0%)	Bdq, Lzd, Cs, Cfz, Pto, Z, Am, Pas, Mfx, Lfx, E, Amx-Clv, Cm	MDR 13–18 months & XDR 24–36 months	21/26 (80.8%)	3/26 (11.5%)	24/26 (92.3%)	2/26 (7.7%)	–	–	–
Koirala et al. (119)	29 countries/ regions	Prospective cohort	As of Jan 31^st^ 2021	883 patients with DR-TB/MDR/RR-TB/XDR-TB (28–49 years)	67/871 (7.7%)	NA	13–23 months	226/383 (59.0%)	58/383 (15.1%)	284/383 (74.2%)	11/383 (2.9%)	25/383 (6.5%)	63/383 (16.5%)	-
Ndjeka et al. (120)	South Africa	Retrospective cohort	2017	688 patients with RR-TB (33–51 years)	493/688 (72%)	Bdq, Lfx or Mfx, Cfz, E, Z, Hh, Eto or Pto	24 months	507/688 (74%)	–	507/688 (74%)	4/688 (1%)	162/688 (24%)	44/688 (6%)	
Zhang et al. (121)	East China	Retrospective cohort	2018–2020	102 patients with RR/MDR/XDR-TB (28–52 years)	–	Bdq, Lfx or Mfx, Cm or Amk, Cs, Pto, Z, Cfz, E or Lzd or Pas	18–20 months	71/102 (69.6%)	23/102 (22.5%)	94/102 (92.2%)	3/102 (2.9%)	1/102 (1.0%)	4/102 (3.9%)	
Fadeyi et al. (122)	Nigeria	Prospective single-arm	2020–2022	20 patients with DR-TB/RR-TB/ Pre-XDR-TB (5–18 years)	1/20 (5%)	Bdq, Dlm, Lzd, Cfz	9 months	11/20 (55%)	3/20 (15%)	14/20 (70%)	–	5/20 (25%)	1/20 (5%)	–
Padmapriyadarsini et al. (27)	India	Prospective cohort study	2019–2021	165 MDR-TBF_Q+_ or/and MDR-TB_SLI+_ (18–56 years)	–	Bdq, Dlm, Lzd, Cfz	24–36 weeks	139/153 (91%)	–	139/153 (91%)	2/153 (1.3%)	4/153 (2.6%)	7^−^/153 (4.5%)	1/153 (0.6%)

TB, Tuberculosis; RR, Rifampicin-resistant; MDR, Multidrug-resistant; Km, Kanamycin; Cm, Capreomycin; Amk, Amikacin; Cfz, Clofazimine; Gfx, Gatifloxacin; Gfx-h, High-dose gatifloxacin; E, Ethambutol; H, Isoniazid; Hh, High-dose isoniazid; Hhm, Medium-high dose isoniazid; Z, Pyrazinamide; Pto, Prothionamide; Mfx, Moxifloxacin; Mfxh, High-dose moxifloxacin; Lfx, Levofloxacin; Cs, Cycloserine; Pmd, Pretomanid; Bdq, Bedaquiline; Dlm, Delamanid; Lzd, Linezolid; Ipm, Imipenem; Mpm, Meropenem; Mpm-Clv, Meropenem-clavulanate; Ipm-clv, Imipenem-clavulanate acid; Cpm, Carbapenems; Trd, Terizidone; PZ, Pasiniazid; Amx, Amoxicillin; Str, Streptomycin; Clr, Clarithromycin. NA, Not available; NS, Not stated; LFT, Lost from treatment; TDT, Total duration of treatment; DM, Diabetes mellitus.

*With DM group; ^+^Without DM group; ^−^Change treatment.

^a^A daily oral regimen of 26 weeks was given to all patients, with the opportunity to prolong it to 39 weeks if their cultures were positive at week 16. Bdq (400 mg once a day for 14 days, then 200 mg 3 times per week thereafter), plus background regimen.

Indeed, numerous clinical studies confirm that the strong bactericidal and sterilizing activity of BDQ-containing regimen originally found in mouse models of TB (123, 124). BDQ has demonstrated potent clinical activity against MDR-and XDR-TB complex strains (27, 118, 121, 122). Importantly, BDQ-containing treatment regimens have showed improved outcomes rate over SLID-containing regimens for the treatment of patients with RR-/MDR-TB (120). Recently, the WHO suggested SLID be replaced in the standard STR with a BDQ-containing regimen. The recommended dose of BDQ is 400 mg orally once daily for 2 weeks followed by 200 mg orally three times weekly for 22 weeks and total duration of BDQ treatment was 24 weeks (117, 118, 120). Many studies have reported the use of BDQ favorable outcomes rates at week 24 of treatment exceeding 80% (15, 117). A very recent study in China has showed that 80.8% of MDR-/XDR-TB patients were cured after receiving BDQ-containing regimens (118). Another recent study from same country reported that the rate of success of culture conversion at 24 weeks is 85.3% of MDR-and XDR-TB patients after receiving BDQ-containing regimens (125). Of note, the findings of this study are more impressive for the treatment of 39 MDR-TB, 56 pre-XDR-TB, and 82 XDR-TB patients, even though for the treatment of pre-XDR and XDR-TB patients are more difficult than MDR-TB patients (125). They have given several feasible explanations for discrepancies in culture conversion rates across studies. Among these, the first one is background regimens which can influence results. For example, LZD can have a good impact on MDR-, pre-XDR- and XDR-TB patient in clinical outcomes. The second one is that BDQ accumulates to comparatively high concentrations in adipose tissue which is confirmed by previous pharmacokinetics research (126). It is interesting to note that MDR-/XDR-TB treatment containing BDQ with compassionate use of LZD showed relatively good success rates (118). Another similar retrospective cohort study reported that the combination of BDQ with LZD and/or imipenem (IPM) showed relatively good success results for treatment of MDR-TB with previously treated extensive and highly resistant TB patients (113). These findings indicate that the combination of BDQ and LZD has good activity in the treatment of MDR-/XDR-TB. Finally, it can be concluded that the administration of BDQ might be associated with additional useful for pre- and XDR-TB patients.

## 12 Resistance mechanisms of BDQ

BDQ is among the last anti-TB drugs approved for the use of MDR-and XDR-TB treatments, which are responsible for reducing mortality rates and improving outcomes (14, 127), and has been in use since 2012. Naturally, the risk of emerging resistance increases due to the widespread use of a new antibacterial drug. As of today, BDQ resistance raises concerns, soon after the introduction for the treatment of MDR-and XDR-TB patients. Indeed, the risk of increasing resistance to BDQ in *Mtb* can be occurred naturally or using for treatment in combination other antibiotics (56). Interestingly, the prevalence of BDQ resistance was 8.9% in isolates resistant to any first- and second-line drug, indicating that the rate of BDQ resistance also rose along with the diversity of drug resistance types and the complexity of resistant background (55). Additionally, retreated patients had a greater rate of BDQ resistance (66.7%) than newly diagnosed patients (33.3%), suggesting this attributed to the previous medical history requires for the use BDQ.

Importantly, the mechanism of BDQ resistance in *Mtb* mainly involves three genes, namely, the *atpE* (128), *Rv0678* (52), and *pepQ* (54) genes. As of today, genetic mutations or resistance-associated variants (RAVs) in the *atpE*, *Rv0678*, *Rv1979c*, and *pepQ* genes have been associated with BDQ resistance (23, 129, 130). The *atpE* gene encodes the ATP synthase by targeting subunit C and its mutations are usually linked with high-level BDQ resistance in *Mtb* but the frequency of mutations is relatively low among TB patients (16). The binding of BDQ to subunit C of ATP synthase can be failure due to genetic mutations or RAVs in the *atpE* genes. Therefore, mutations in the *atpE* genes (A28P, A28V, G61A, A63P, and I66M) are associated with high-levels resistance (10 to 128-fold MIC) to BDQ. Furthermore, a report demonstrated that the frequency of *atpE* gene mutations in TB patients is extremely low, they are responsible for high-level of BDQ resistance (111).

Mutations in *Rv0678*, which codes for the repressor of the efflux pump MmpL5-MmpS5, are the primary cause of BDQ resistance and typically result in low-level resistance (131). Importantly, a very recent study reported from Chongqing, China that BDQ resistance was mostly caused by mutations in the *Rv0678* gene, with the most frequent mutation type being A152G (55). Mutations in the *Rv0678* gene encoding the efflux pumps MmpS5-MmpL5 as well as the intergenic region between Rv0678 and MmpS5 were also revealed to be the cause of BDQ resistance (132). Of note, different types of mutations in the *Rv0678* gene conferring resistance to BDQ derived clinical and *in vitro* isolates of *Mtb* are summarized in Figures 2, 3 (8, 16, 22, 55, 56, 87, 90–93). In 2016, the gene *pepQ* (*Rv2535c*) was discovered as potential which may be associated to BDQ resistance (54). BDQ resistance has been associated to mutations in the genes *pepQ* and *Rv1979c*, which encodes a potential Xaa-Pro amino-peptidase and a putative permease, however the underlying mechanisms are yet unknown (111, 130). A study showed that mutations in *pepQ* gene have low-level resistance (up to 4 fold) to BDQ in mice (54). However, reduced antimycobacterial susceptibility to BDQ has been reported to be associated with mutations in *Mtb* strains in the *Rv0678*, *pepQ*, and *atpE* genes (90).

Nevertheless, a portion of BDQ resistant isolates identified no mutations in *Rv0678*, *atpE*, and *pepQ* in *in vitro* and clinical settings, although mutations in *Rv0678*, *atpE*, and *pepQ* confer major resistance to BDQ, suggesting that there are other unknown mechanisms of resistance to BDQ (133). Importantly, a new gene, *glpK*, (an insertion G572 mutation in *glpK*) is identified that resulted in a frame shift and loss of function, leading resistance to BDQ (93). The enzyme glycerol kinase (GlpK), encoded by *Rv3696c*, is a key enzyme for the glycerol uptake and metabolism. It catalyzes the phosphorylation glycerol to glycerol-3-phosphate for glycerophospholipids synthesis (134, 135) or the synthesis of glycolysis and gluconeogenesis (136). Further studies are required to explore the exact role of *glpK* gene. Finally, it can be concluded that resistance-conferring mutations in the *atpE*, *Rv0678* and *pepQ* genes might be potential diagnosis determinants. In addition, careful evaluation is recommended for the prescription of BDQ in the regimen for the treatment of DR-, MDR-and XDR-TB patients. Although BDQ has been shown to be extremely effective in the treatment of MDR-TB until this point (55), but misdiagnosis, insufficient and/or incomplete use may lead to the emergency of resistance to *Mtb* strains (19, 92). For instance, patients infected with an MDR outbreak strain in Eswatini and South Africa those carried the RR variant I491F in the *rpoB* gene continued to receive RIF drug despite it being ineffective because this variant was not identifiable by conventional phenotypic or genotypic testing (137). Therefore, it is important to dynamically assess the BDQ resistance for optimizing BDQ administration regimen, furthermore to prevent the emergence of acquired resistance and maximize the efficacy of new drug.

## 13 Mechanisms of cross-resistance to CFZ and BDQ

Cross-resistance is a form of resistance to all drugs in the same class resulting from a single mechanism. Drugs that belong to the same class typically share a chemical structure, which means they act on the same cell target and can cause cross-resistance. Resistance to the CFZ and BDQ almost always arises from the build-up of mutations in the chromosomal genes that control permeability, active efflux, and drug targets. The presence of resistance-associated mutations linked to *Rv0678* resulted in increased MICs for BDQ and CFZ in murine isolates (2 to 8 fold and 2 to 4 fold, respectively), and 2 to16 fold MICs for BDQ in clinical isolates. Clinicians are increasingly choosing BDQ and CFZ to treat DR-TB in recent years. Both drugs impair the energy metabolism in mycobacteria and several studies have reported cross-resistance between CFZ and BDQ (8, 50, 130, 137–140). Drug resistance for these two drugs should be closely monitored given their critical role in the treatment. The existence of cross-resistance between new and old drug used in MDR-TB strains undermines using new ones effectively. Therefore, cross-resistance to CFZ and BDQ thus emerges as a significant concern that possibly undermines the efficacy of BDQ treatment for DR-TB. Notably, 12/13 (92.31%) of the *Mtb* clinical isolates presented resistance to BDQ when the MIC of CFZ was ≥4 mg/L. (50) In addition, half of the CFZ-resistant isolates were classified as BDQ-resistant when the breakpoint for BDQ (0.12 mg/L) was used. This result was similar with other studies (8, 21), which revealed that at least half of the CFZ-resistant *Mtb* isolates were still sensitive to BDQ. However, every single isolate that was BDQ-resistant was also CFZ-resistant. Another important study reported that 12% of the MDR-TB patients had resistance to both BDQ and CFZ (45). According to clinical characterization, CFZ-resistant TB patients were more likely to develop BDQ resistance due to prior CFZ or BDQ exposure as well as pre-XDR-TB (50, 141). Conversely, it is speculated 4.4% MDR-TB isolates resistant to BDQ without record prior use of BDQ, suggesting this promising new drug could be rapidly lost due to the emergency of BDQ resistant isolates, although BDQ demonstrated remarkable effectiveness against MDR-TB strains (55).

Interestingly, MmpS5 and MmpL5, which are bacterial membrane proteins, are part of the efflux pump system shared by both BDQ and CFZ and exposure to CFZ may promote efflux-based resistance, leading to cross-resistance between these two drugs (52, 130). Many different studies have documented that mutation in *Rv0678* can cause cross-resistance between BDQ and CFZ in *Mtb* strains (50, 142). Significantly, various mutations in the *Rv0678* gene associated with cross-resistance to BDQ and/or CFZ derived clinical and *in vitro* isolates of *Mtb* are summarized in Figures 2, 3. *Rv0678* RAVs enhanced the CFZ and BDQ MICs in murine isolates by 2- to 4-fold and 2- to 8-fold, respectively, and increased the BDQ MICs in clinical isolates by 2- to 16-fold (23, 131). Numerous studies have confirmed that CFZ exposure in the past promotes BDQ resistance linked to mutations in *Rv0678* and *pepQ* genes (52, 54). On the other hand, a current study demonstrated that CFZ resistance developed following the sole administration of BDQ. Interestingly, while the other two cases lacked any known mutations linked to CFZ and BDQ resistance, one out of three cases harbored a genetic mutation at the *Rv0678* locus (8). A recent study supported the potential of cross-resistance by demonstrating mutations L117R (T350G) and M146T (T437C) in *Rv0678* and R409Q (G1226A) in *Rv1979c* in the strains *Mtb* resistant to both drugs BDQ and CFZ (86). The findings of the study suggest that more mechanism studies, i.e., whole genome sequencing, are needed to discover cross-resistance-related novel mechanisms in the BDQ and CFZ against *Mtb* strains (86). Significantly, clinical isolates rarely contain mutations in the BDQ target gene, *atpE*, and the majority of strains that exhibit phenotypic resistance to BDQ have mutations in the non-target *Rv0678* gene. Reduced susceptibility to BDQ is linked to mutations in the *Rv0678* and *atpE* genes, which have been found in both clinical isolates and strains that were chosen *in vitro*. There are few clinical studies reported to BDQ against *Mtb* strains that may be a possible reason to see mutations in laboratory BDQ-resistant *Mtb* strains *in vitro* that are very different from those isolated from mice or clinical isolates. Indeed, misdiagnosis can promote the spread of particular MDR *Mtb* strains through selection and subsequent transmission (92). Additionally, recent investigations showed that mutations in *Rv0678* emerged that were linked to BDQ/CFZ resistance due to treatment failure and/or poor outcomes in MDR-TB patients.

## 14 Pharmacokinetics of BDQ

Compared to the data available for other anti-TB drugs, BDQ has fewer pharmacokinetics data published in the literature. Drug exposure in the case of BDQ is associated with body weight, age, race, albumin, and concurrent RIF use (143). The concentration of BDQ is significant for activity that has been shown to be concentration-based where the high BDQ concentrations were linked to a quicker reduction in mycobacterial load for patients within 24 weeks and culture conversion of sputum after 6-month treatment (87, 144). Therefore, it is crucial to know the optimal BDQ exposure for improvement of MDR-TB treatment. The recommended dosage of BDQ for adult patients is 400 mg daily for 2 weeks, then 200 mg three times a week for an additional 22 weeks (145). Surprisingly, drug susceptibility is a significant factor influencing the sputum culture conversion, as earlier stated (8). Of note, a current study by Shao et al. (146) demonstrated that the 24-h area under the curve (AUC_0-24h_/MIC) higher than 175.5 showed an increased probability of sputum culture conversion following a two-month therapy. In addition, after 6 months of treatment, those with AUC_0-24h_/MIC values higher than 118.2 demonstrated a higher likelihood of sputum culture conversion and AUC0-24 h/MIC higher than 74.6 showed a higher probability of a favorable outcome after treatment.

Importantly, the relative absorption of BDQ was found to increase around 2-fold when the drug is administered along with food compared to without food or fasting conditions, and consequently, it was advised to take it with food (147). The BDQ pharmacokinetic profile reveals that the maximal serum concentration (C_max_) is reached about 5 h after delivery, and the curative half-life is about 24 h following 2 weeks of 400 mg daily therapy. Therefore, it is advised to take BDQ with food. A very current study suggested that probability of target attainment (PTA) declines when patient body weight rises for both BDQ and PMD (148). An increase in BDQ dosage may not be practical for all individuals because the existing BDQ dose regimen is linked to safety hazards of the Fridericia-corrected QT (QTcF) prolongation and hepatic side effects. Further, bodyweight-based dose optimization for BDQ may be effective for assessing efficacy and safety.

## 15 Conclusion and perspectives

Our current review provides information on the efficacy of BDQ and CFZ alone and their combination use in the treatment of DR-/MDR-TB patients. The addition of BDQ and CFZ to the therapeutic TB regimens significantly improves results for MDR-TB patients. The use of BDQ and/or CFZ-containing regimens demonstrated low mortality with high culture conversion rates. Along with this, our review also supports and provides information regarding drug resistance as well as DST to BDQ and CFZ in *in vitro* and clinical *Mtb* strains. The second-line anti-TB drugs without DST lead to the potential for known and unknown drug resistance to the BDQ and CFZ. BDQ resistance is influenced by prior use of CFZ among CFZ-resistant patients. Drug resistance significantly reduces treatment efficiency, suggesting that phenotypic and genotypic tests are required before using BDQ and CFZ alone or their combination for the treatment of MDR-TB patients. The goal of TB drug development and discovery is to produce TB drugs and regimens that are better than those on the market today in terms of their accessibility, ease of use for all patient populations, efficacy, mechanisms of action and resistance. This review summarizes above all issues for BDQ and CFZ against *Mtb* strains, which may aid in the development and discovery of novel anti-TB drugs and even other drug combinations. TB control programs desperately need novel drugs that are as effective against MDR/XDR strains of *Mtb* while also having the advantage of being easier to administer and having shorter treatment duration. In the field of TB, the biochemical, target-driven approach to drug development has mainly been abandoned in favor of whole-cell or target based whole-cell screening methods. Additionally, this method produced a number of new, chemically verified targets that are currently useful for compound optimization based on targets. It is interesting to note that metabolism of energy and cell envelope biosynthesis seems to be heavily impacted by these novel anti-mycobacterial drugs, indicating that those metabolic regions are particularly susceptible or accessible. Therefore, an in-depth comprehension of the clinical efficacy, DST, mutations associated with individual resistance and cross-resistance, and pharmacokinetics of CFZ and BDQ against *Mtb* can offer fresh perspectives on how to enhance treatment outcomes, lower mortality, avoid drug resistance, and stop the spread of TB. Additionally, it will support the creation of quick molecular testing techniques and innovative TB drugs.

## Author contributions

MI: Conceptualization, Investigation, Visualization, Writing – original draft. MA: Data curation, Writing – original draft. ZL: Data curation, Writing – original draft. MK: Data curation, Writing – original draft. BY: Data curation, Writing – original draft. HH: Investigation, Writing – original draft. XT: Investigation, Visualization, Writing – original draft. CC: Investigation, Visualization, Writing – original draft. RB: Writing – review & editing, Formal analysis, Resources. HA: Writing – review & editing, Formal analysis, Resources. XZ: Writing – review & editing, Formal analysis, Resources. SK: Writing – review & editing, Data curation, Investigation. CF: Writing – review & editing, Data curation, Investigation. CL: Writing – review & editing, Data curation, Investigation. SH: Writing – review & editing, Investigation. ST: Writing – review & editing, Investigation. NZ: Writing – review & editing, Investigation. JH: Writing – review & editing, Conceptualization, Funding acquisition, Project administration. TZ: Conceptualization, Funding acquisition, Project administration, Writing – review & editing.
